# The effect of gut microbiome on tolerance to morphine mediated antinociception in mice

**DOI:** 10.1038/srep42658

**Published:** 2017-02-17

**Authors:** Minho Kang, Ryan A. Mischel, Sukhada Bhave, Essie Komla, Alvin Cho, Charity Huang, William L. Dewey, Hamid I. Akbarali

**Affiliations:** 1 Department of Pharmacology and Toxicology, Virginia Commonwealth University, Richmond, VA 23298, USA.

## Abstract

There is growing appreciation for the importance of gastrointestinal microbiota in many physiological and pathophysiological processes. While morphine and other narcotics are the most widely prescribed therapy for moderate to severe pain clinically, they have been noted to alter microbial composition and promote bacterial translocation to other tissues. Here we examined the pharmacodynamic properties of chronic morphine in mice following bacterial depletion with oral gavage of an antibiotic cocktail (ABX). ABX significantly reduced gut bacteria and prevented chronic morphine induced increases in gut permeability, colonic mucosal destruction, and colonic IL-1β expression. In addition, ABX prevented the development of antinociceptive tolerance to chronic morphine in both the tail-immersion and acetic acid stretch assays. Morphine tolerance was also reduced by oral vancomycin that has 0% bioavailability. These findings were recapitulated in primary afferent neurons isolated from dorsal root ganglia (DRG) innervating the lower gastrointestinal tract, wherein *in-vivo* administration of ABX prevented tolerance to morphine-induced hypoexcitability. Finally, though ABX repeatedly demonstrated an ability to prevent tolerance, we show that it did not alter susceptibility to precipitation of withdrawal by naloxone. Collectively, these finding indicate that the gastrointestinal microbiome is an important modulator of physiological responses induced by chronic morphine administration.

There is growing appreciation for the importance of gastrointestinal microbiota in many physiological and pathophysiological processes. Under normal conditions, the human and mouse gut are largely colonized by Bacteroidetes and Firmicutes, with several other phyla present in smaller proportions^1^. Epithelial tight junctions and continuous gastrointestinal (GI) immune surveillance contain these bacteria within the gut lumen, halting access to host tissues and circulation. Compromise of bacterial composition and/or localization (dysbiosis) has been noted to play a significant role in pathogenesis. This includes gastrointestinal pathologies such as irritable bowel syndrome (IBS) and inflammatory bowel diseases (IBD), and disorders of neural origin such as anxiety and depression^2^,^3^. Inversely, recent studies have also highlighted the ability of probiotics to reduce visceral pain in conditions such as IBS^4^,^5^. For example, administration of *Lactobacillus acidophilus* reduces visceral hypersensitivity to colorectal distension in an IBS rodent model. While the mechanisms remain unclear, associated changes in μ-opioid receptor (MOR) expression, as well as in the electrophysiological properties of sensory neurons within the myenteric ganglia have been demonstrated^5^. Furthermore, bacterial proteins can directly enhance the excitability of nociceptors in dorsal root ganglia^6^. These findings collectively suggest that gastrointestinal microbiota may modulate pain pathways by a mechanism involving opioid signaling. In this context, recent studies by Meng *et al*.^7^ demonstrate that chronic morphine exposure in mice induces alterations of bacterial composition and compromises GI epithelial tight junction integrity, promoting secondary gut wall inflammation. Indeed, clinical studies have suggested that narcotic use escalates disease severity in patients with Crohn’s disease (CD), and may be associated with increased recurrence from remission^8^. In addition, data from the TREAT registry, comprised of over 5,000 patients with CD, suggest that narcotic use was associated with a 1.5-fold increase in risk of mortality and 3-fold increase in risk of infection compared to patients not taking opioid analgesics^9^. Thus, the effect on pain pathways, particularly sensory neurons, raises the question of whether the gut microbiome is linked to effects of chronic morphine, including the development of tolerance and dependence. The phenotypic and physiological changes that occur following depletion of gut bacteria by broad-spectrum antibiotics parallel those observed in axenic (germ-free) mice. For instance, Anitha *et al*.^10^ demonstrated a loss of nitrergic and total neurons by both methodologies. Reikvam *et al*.^11^ observed similar alterations of gene expression in intestinal epithelial cells of antibiotic-induced depletion of gut bacteria as in germ-free mice. In the present study, we examined whether the depletion of the gastrointestinal microbiome alters the pharmacodynamic properties of chronic morphine treatment, including destruction of colonic mucosa, development of tolerance to antinociception and sensory neuron hypoexcitability, and formation of dependence.

## Results

### Chronic morphine compromises gut epithelial barrier function and increases gut permeability

**Figure 1:**
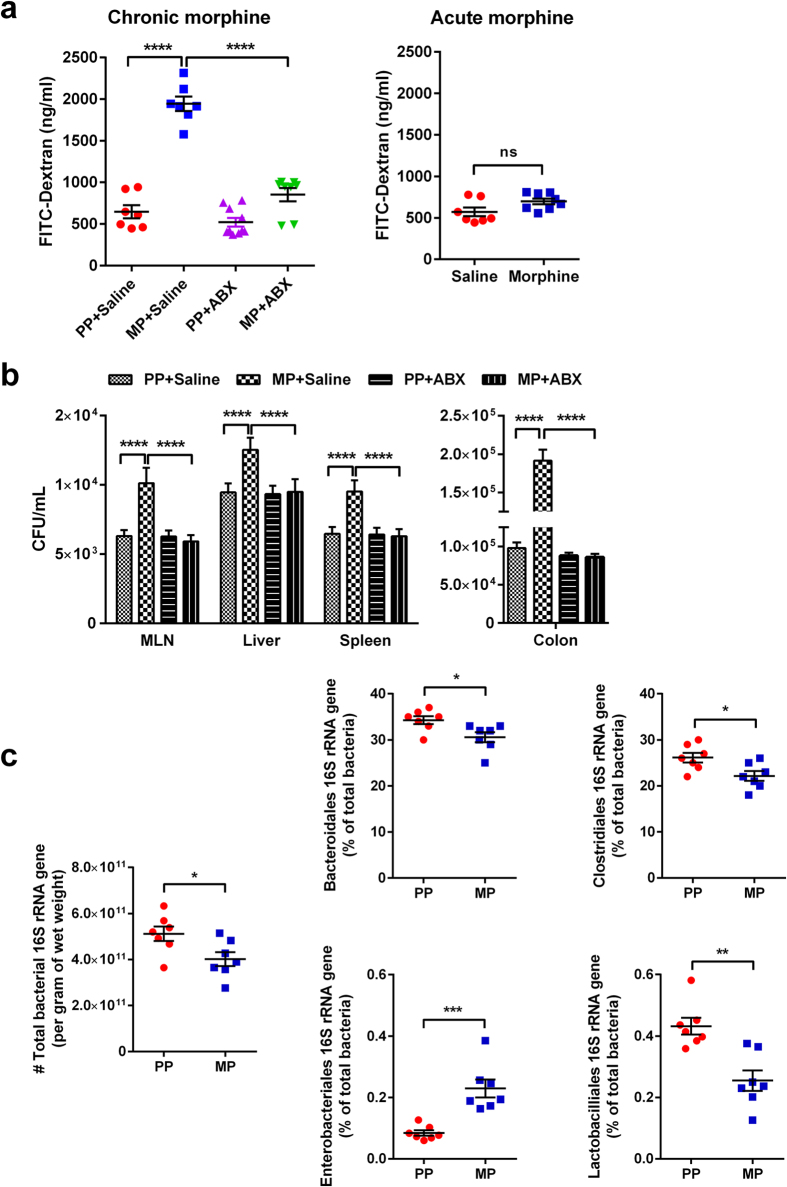
Morphine increases gut epithelial permeability. (**a**) Fluorometric quantification of FITC from whole-blood samples in each treatment group. Antibiotic treatment *per* se did not significantly alter FITC-dextran concentration in placebo-pellet mice. Chronic morphine treatment resulted in an increase in blood FITC-dextran concentration, which was mitigated by the addition of antibiotics. Acute morphine did not alter blood FITC concentration compared to saline. N = 7, ****p < 0.0001 by two-way ANOVA with Bonferroni post-hoc analysis (*left panel*). N = 7, ns not significant by unpaired t-test (*right panel*). (**b**) Quantification of colony-forming units (CFU) on blood agar plates seeded with mesenteric lymph node (MLN), liver, spleen, and colon tissue samples from each treatment group. Chronic morphine treatment significantly increased bacterial populations in all tissues tested. N = 6, ****p < 0.0001 by two-way ANOVA with Bonferroni post-hoc analysis. (**c**) Total bacterial 16 S rRNA gene copy number and order analysis in PP and MP mice as determined by qRT-PCR. Relative bacterial order abundances were determined as a relative percentage of total bacteria in fecal pellets. Mice treated with chronic morphine demonstrate reduced total bacterial abundance, reduced relative abundances of bacteroidales, clostridiales, and lactobacilliales, and increased relative abundance of enterobacteriales when compared to PP controls. N = 7, *p < 0.05, **p < 0.01, and ***p < 0.001 by unpaired t-test.

**Figure 2:**
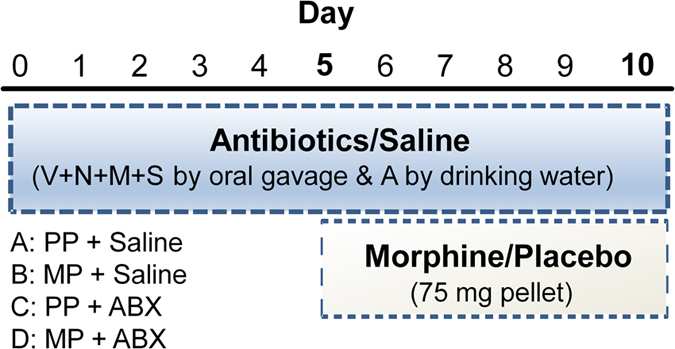
Protocol for administration of antibiotics and morphine. Protocol schematic for oral gavage administration of vancomycin (V, 5 mg/kg), neomycin (N, 10 mg/kg), metronidazole (M, 10 mg/kg), and streptomycin (S, 10 mg/kg) every 12 hours with ampicillin (1 g/L) by drinking water. Morphine or placebo pellets were implanted on day 5 and tolerance tested on day 10.

**Figure 3:**
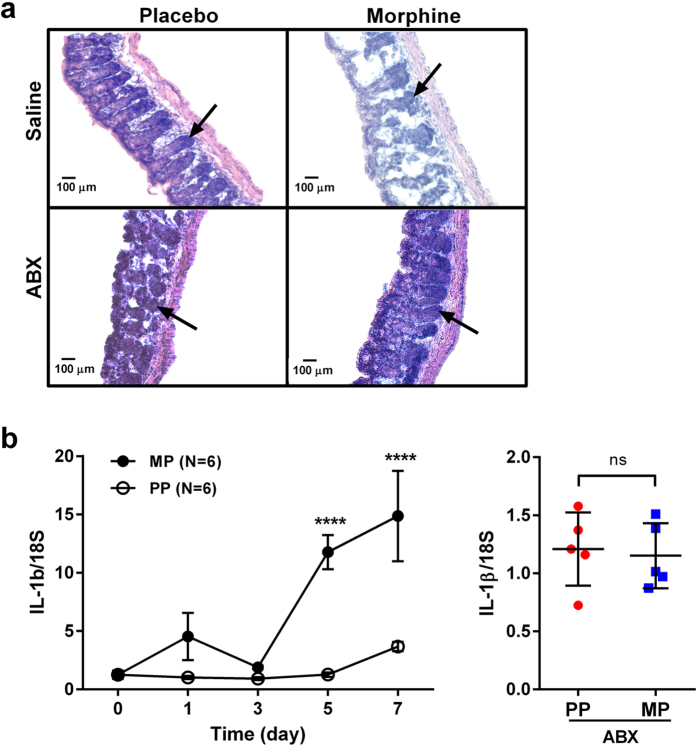
Gut bacteria depletion prevents chronic morphine-induced mucosal damage and IL-1β expression. (**a**) H&E staining of colon sections demonstrates mucosal damage with chronic morphine exposure, which was abated by antibiotic treatment. Only minor mucosal changes were noted with antibiotic treatment *per se*. Arrow indicates mucosal crypts. (**b**) Chronic morphine significantly increases colonic IL-1β mRNA expression at 5 and 7 days post pellet implantation (*left panel*). N = 6 for each sample day, ****p < 0.0001 by two-way ANOVA with Bonferroni post-hoc analysis. This increase in expression is prevented by ABX treatment (*right panel*) measured at day 5 following pellet implantation. N = 5, *p < 0.05 by two-way ANOVA with Bonferroni post-hoc analysis.

**Figure 4:**
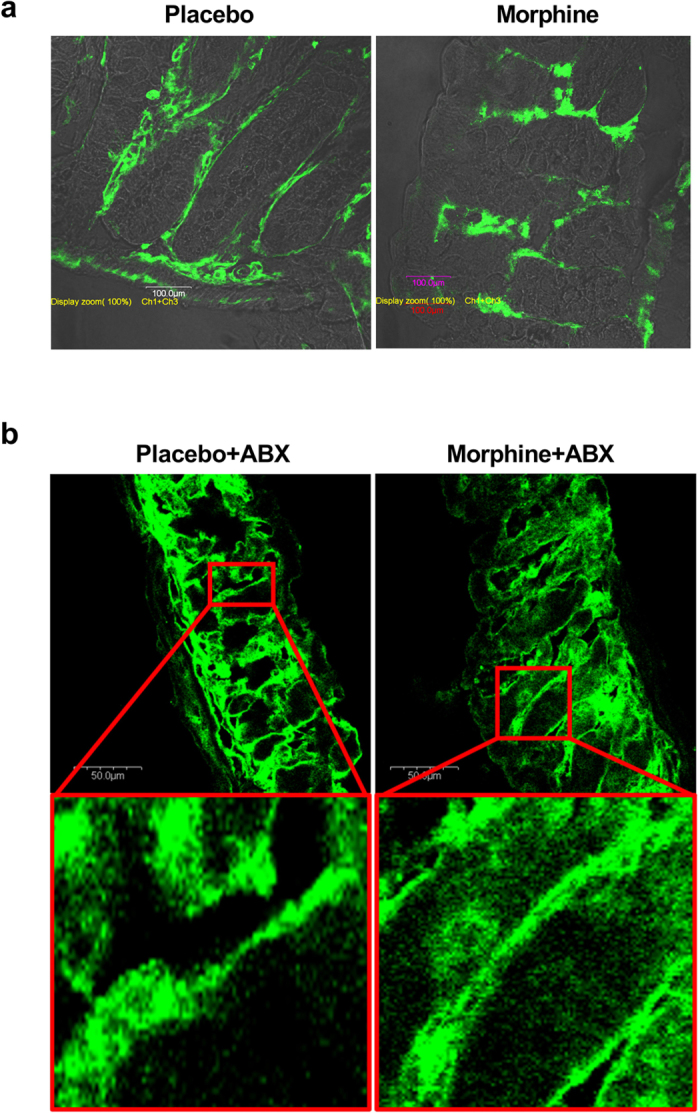
Effect of antibiotics on tight junction organization and mRNA expression with chronic morphine. (**a**) Representative immunohistochemical staining of colon sections from placebo-pelleted and morphine-pelleted mice. Occuludin staining in green is superimposed on phase contrast image. Scale bar: 100 μm. (**b**) Colon cross-section from ABX treated placebo pellet and morphine pelleted mice. Boxed regions correspond to higher-magnification images in the insets. Scale bar: 50 μm.

To study the effect of chronic morphine on gut permeability, serum FITC concentrations were measured following oral administration of FITC-dextran (Fig. 1a). Five days after pellet implantation, FITC concentration in serum was significantly greater in morphine-pelleted (MP) mice than in placebo-pelleted (PP) controls (p < 0.0001). The increase in gut permeability by chronic morphine was significantly reduced in mice treated with broad-spectrum antibiotics (ABX), as described in Methods and shown in Fig. 2. No increase in permeability was observed with acute morphine treatment (10 mg/kg, s.c.) or saline. To determine the effects of chronic morphine on bacterial translocation, colon, mesenteric lymph node (MLN), liver, and spleen tissue samples were collected and cultured on blood agar plates (BD Biosciences) for 24–48 hours and colony forming units (CFUs) quantified. In morphine-pelleted mice, the number of bacterial colonies in all tissues was significantly higher than in PP controls (p < 0.0001, Fig. 1b). This bacterial translocation was significantly reduced in mice treated with ABX. The total bacterial load in fecal samples of MP mice was slightly but significantly reduced as determined by 16 S rRNA gene copy numbers (p < 0.05, Fig. 1c). An order analysis indicated that Bacteroidetes (Bacteriodales) and Firmicutes (Clostridiales and Lactobacillales) were significantly reduced in the fecal samples from MP mice, while Proteobacteria (Enterobacteriales) were enhanced. Histological examination of hematoxylin and eosin (H&E) stained colon samples showed disruption of mucosal crypts and lamina propria in MP mice that was not noted in PP mice (Fig. 3a). Treatment with ABX resulted in structural changes in both placebo- and morphine-pelleted mice, with an apparent alleviation of mucosal damage with chronic morphine. In conjunction with enhanced bacterial translocation due to increased gut permeability and microbial dysbiosis, a temporally dependent increase in colonic mRNA expression of IL-1β was noted in MP mice. The expression of IL-1β was significantly enhanced in the colon of MP mice after 5 days. The increased expression of IL-1β was prevented by ABX treatment when examined on day 5 (p < 0.05, Fig. 3b). The effect of chronic morphine on the localization of the tight junction protein occludin was determined by immunohistochemistry in cross sections of the colon. Occludin staining was localized mainly to the epithelial junctions in placebo-pelleted mice. Chronic morphine treatment resulted in disorganization of the tight junction in the colon (Fig. 4a), similar to the observations in the small intestine^7^. ABX treatment appeared to preserve occludin staining at the tight jucntions in the MP colon with less disorganization. The mRNA expression of occludin and ZO-1 remained unchanged with chronic morphine, however surprisingly there was a significant increase with ABX treatment in MP colon (Supplemental Data). These findings indicate that chronic morphine-induced disruption of the epithelial integrity allows for enhanced permeability, bacterial translocation and inflammation. These effects appear to be prevented by ABX treatment.

**Figure 5:**
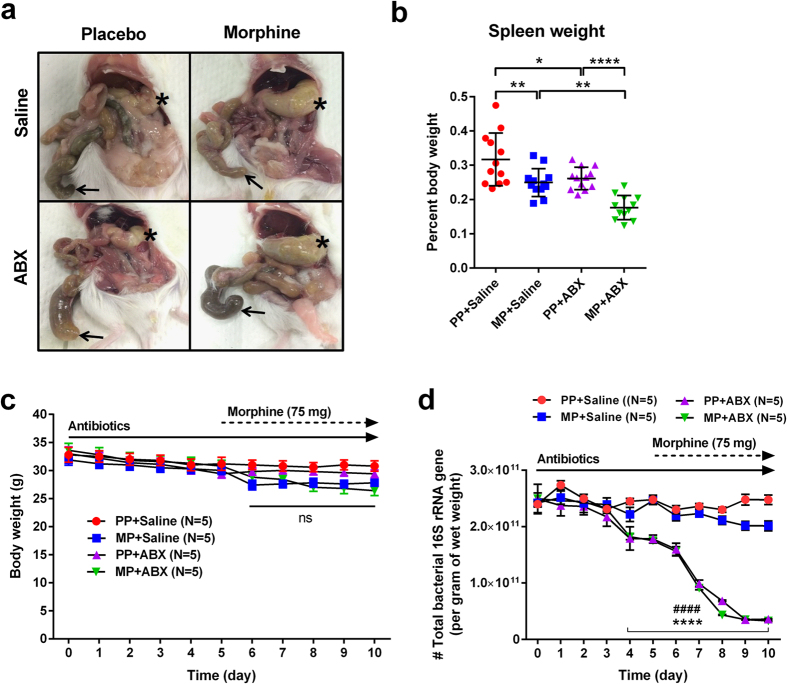
Assessment of gut microbiota, spleen, cecum and body weight in morphine-pelleted mice following antibiotic treatment. (**a**) Post-mortem necropsy images demonstrating antibiotic-induced cecal bloating (*arrows*) and morphine-induced gastric distention (*asterisks*). (**b**) Spleen weight measurements on day 10 for each treatment cohort. Antibiotic treatment produced a significant main effect in reducing spleen weight. N = 12, *p < 0.05, **p < 0.01, and ****p < 0.0001 by two-way ANOVA with Bonferroni post-hoc analysis. (**c**) Daily body weight measurements for each treatment group. No significant decrease in body weight was noted with ABX treatment or chronic morphine. N = 5, ns not significant by two-way repeated-measures ANOVA with Bonferroni post-hoc analysis. (**d**) Total bacterial 16 S rRNA gene copy number in fecal samples from all treatment groups. Antibiotic treatment produced significant reduction of bacterial load by day 4 of treatment, and nearly 90% reduction by day 10. N = 5, ****p < 0.0001 (PP + SAL vs PP + ABX) and ^####^p < 0.0001 (MP + SAL vs MP + ABX) by two-way repeated-measures ANOVA with Bonferroni post-hoc analysis.

The efficacy of ABX treatment was evidenced by gross morphological changes indicative of bacterial clearance, i.e. cecal enlargement (*arrows*, Fig. 5a) and splenic atrophy (Fig. 5b). MP mice expressed significant gastric distension (*asterisks*, Fig. 5a) and marginal weight loss (~10%, Fig. 5c) in both ABX- and saline (SAL)-treated groups. ABX treatment had no impact on body weight. Total bacterial abundance was quantified by qRT-PCR amplification of the 16 S rRNA gene in DNA samples isolated from fecal boli. Total bacterial load was significantly reduced by ABX at day 10 (p < 0.0001, Fig. 5d). Examined over the time course of ABX treatment, bacterial abundance began decreasing on day 4, with siginificant depletion observed on day 8.

### Reduction of gut bacteria prevents antinociceptive tolerance to chronic morphine

**Figure 6:**
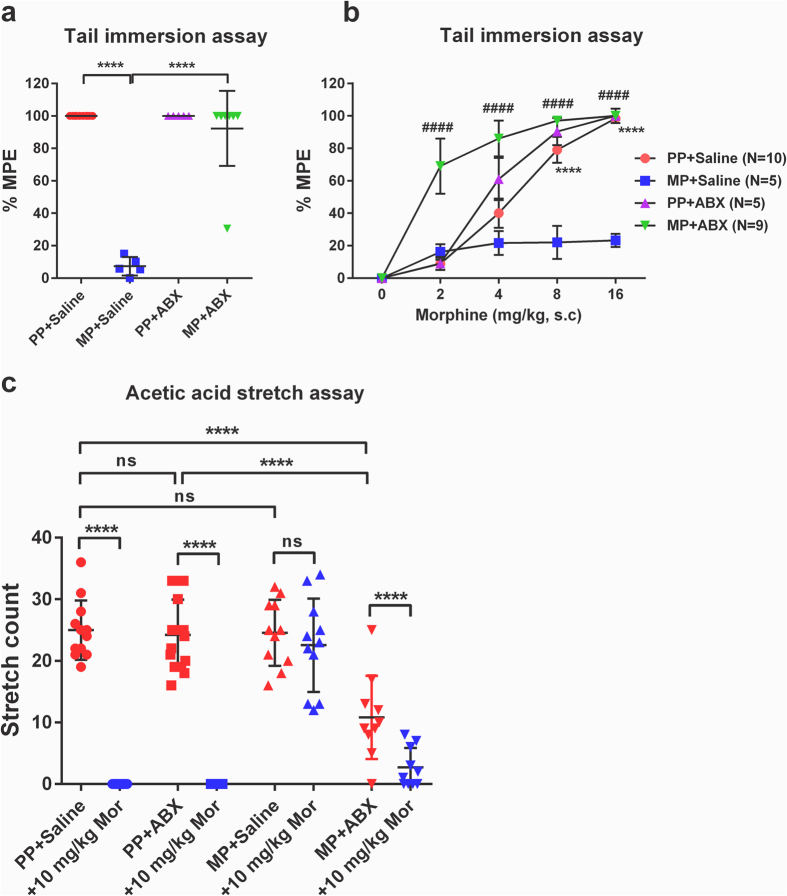
Antibiotic treatment prevents morphine antinociceptive tolerance. (**a**) Chronic morphine exposure resulted in antinociceptive tolerance to morphine challenge (10 mg/kg) that was prevented by ABX in the tail-immersion assay. Results are expressed as the percentage of maximum possible effect (%MPE ± SEM) in response to morphine challenge. N = 6, ****p < 0.0001 by two-way ANOVA with Bonferroni post-hoc analysis. (**b**) Dose-dependence of morphine antinociception in the tail-immersion assay. Ramp morphine administration (2, 4, 8 and 16 mg/kg, s.c.) demonstrated a reduction of efficacy with chronic morphine that was prevented by ABX treatment. PP + SAL (N = 10), PP + ABX (N = 5), MP + SAL (N = 5), MP + ABX (N = 9), ****p < 0.0001 (PP + SAL vs PP + ABX), ^####^p < 0.0001 (MP + SAL vs MP + ABX) by two-way repeated-measures ANOVA with Bonferroni post-hoc analysis. (**c**) Chronic morphine exposure resulted in antinociceptive tolerance to morphine challenge that was prevented by ABX in the acetic acid stretch assay. PP + SAL (N = 12), PP + ABX (N = 15), MP + SAL (N = 11), MP + ABX (N = 10), ns not significant, ****p < 0.0001 by two-way ANOVA with Bonferroni post-hoc analysis.

**Figure 7:**
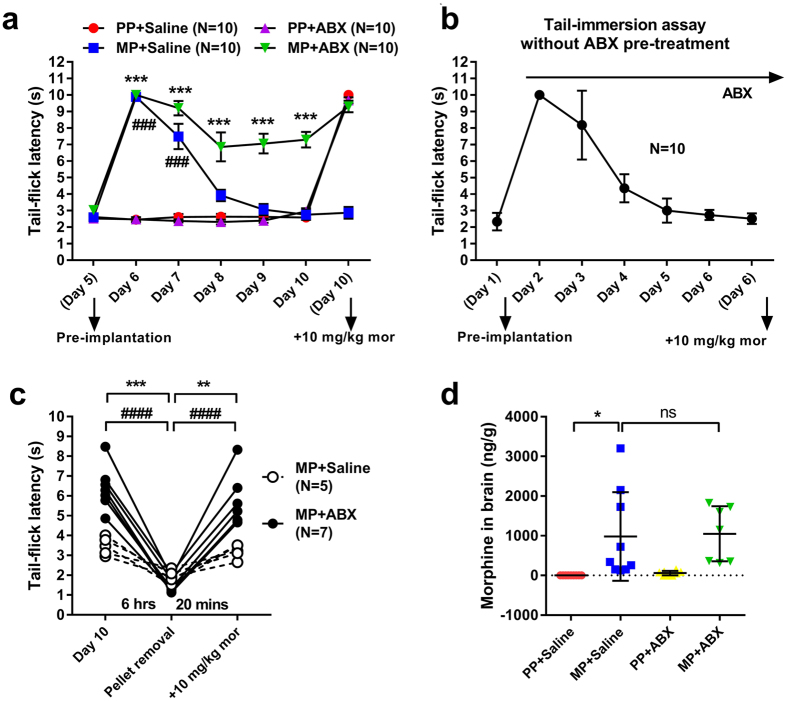
Time course of morphine tolerance in the presence of antibiotics. (**a**) Daily baseline tail-flick latency for each treatment group. Chronic morphine results in antinociceptive tolerance at 48 hr post pellet implantation that is prevented by ABX treatment. Chronic morphine also prevents response to morphine challenge on day 10, which is preserved by ABX treatment. N = 10, ***p < 0.001 (MP + ABX vs PP + SAL), ^###^p < 0.001 (MP + SAL vs PP + SAL) by two-way repeated-measures ANOVA with Bonferroni post-hoc analysis. (**b**) Elimination of the 5-day ABX pre-treatment phase is insufficient to prevent tolerance with chronic morphine. N = 10. (**c**) Morphine pellets removed on day 10 from MP + SAL and MP + ABX groups produces withdrawal regardless of ABX treatment, and implicates morphine responses in tail-immersion observations. MP + SAL (N = 5), MP + ABX (N = 7). (**d**) Brain morphine concentration on day 10 in all treatment groups. Morphine concentrations are increased significantly by morphine pelleting (0.00 ± 0.00 ng/g vs 982.88 ± 372.13 ng/g), but not affected by ABX treatment. PP + SAL (N = 9), MP + SAL (N = 9), PP + ABX (N = 8), MP + ABX (N = 7), ns not significant, *p < 0.05 by two-way ANOVA with Bonferroni post-hoc analysis.

The effect of reducing gut bacteria with ABX on the development of morphine antinociceptive tolerance was examined in the tail-immersion assay. Figure 6a shows the antinociceptive effect of morphine challenge (presented as maximal possible effect) 5 days following pellet implantation and corresponding to day 10 of saline and antibiotic treatment. Morphine challenge produced maximal antinociception in both PP groups. This response was mitigated in MP + SAL mice, but preserved in MP + ABX mice. Figure 6b demonstrates the dose-dependence of these responses. ABX treatment produced no significant effect in PP controls. Morphine tolerance occurred in MP + SAL mice, but not in MP + ABX mice which showed greater sensitivity. Antinociceptive tolerance was also examined in the acetic acid stretch assay. As shown in Fig. 6c, morphine challenge (10 mg/kg, s.c.) produced complete abolition of stretching in both PP cohorts. Concurring with tail-immersion data, this response was mitigated in MP + SAL mice, but preserved in MP + ABX mice. The time course for the development of tolerance was measured by observing baseline tail-flick latencies on each day of treatment. As shown in Fig. 7a, the latency was significantly higher as expected in both MP groups on day 1 after pellet implantation. This effect had diminished in MP + SAL mice at 48 hr after implantation (day 8), but remained in MP + ABX mice through the duration of the study (day 10). To confirm that tolerance had developed, a morphine challenge was given on day 10. All groups responded with an increase in tail-flick latency, except MP + SAL. To examine for duration-dependent effects of ABX, the tail-immersion assay was conducted on mice beginning ABX treatment on the same day as MP implantation (i.e. lacking the 5-day pre-treatment). Figure 7b demonstrates that this truncated regimen was insufficient to observe the higher baseline latencies and preservation of response to morphine challenge demonstrated by mice receiving the full 10-day ABX regimen. These data support the notion that reducing gut bacteria prevents of the development of tolerance to morphine.

In order to determine whether the lack of tolerance in the presence of ABX treatment results from enhanced bioavailability of morphine, pellets were removed and baseline tail-flick latency was determined 6 h after removal of the pellet. As shown in Fig. 7c, baseline latencies converged to 1.69 ± 0.12 s in both SAL- and ABX-treated mice. Subsequent administration of acute morphine challenge (10 mg/kg, i.p.) after 20 min returned tail-flick latency to values observed prior to pellet removal for each group. These findings suggest that morphine tolerance persists in the absence of the pellet while tolerance does not develop in mice that were exposed to antibiotics.

To assess whether antibiotic treatment alters brain morphine concentration, whole-brain samples were collected from all groups and analyzed by gas chromatography/mass spectrometry (GC-MS). Brain morphine concentrations in SAL-treated mice (982.8 ± 372.1 ng/g) did not significantly differ from those of ABX-treated mice (1049.8 ± 244.9, Fig. 7d).

**Figure 8:**
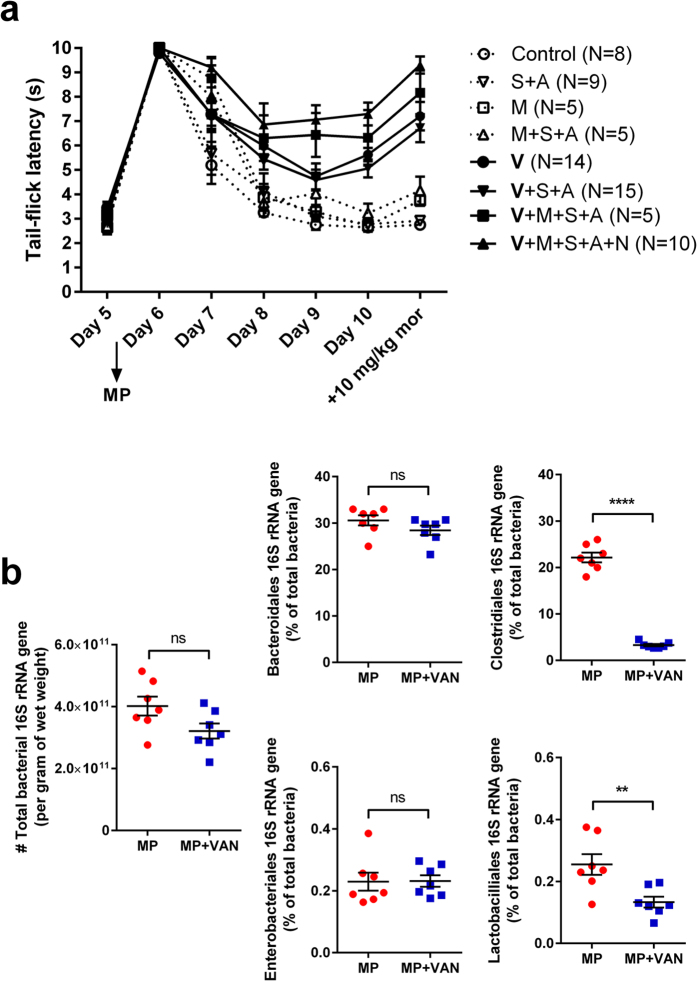
Effect of various combinations of antibiotics on morphine tolerance. (**a**) Daily baseline tail-flick latency for various combinations of vancomycin (V), metronidazole (M), streptomycin (S), ampicillin (A), and neomycin (N). Closed symbols represent significant difference from control by two-way repeated-measures ANOVA with Bonferroni post-hoc analysis. Morphine pellets were implanted on Day 5. Antibiotics were delivered by oral gavage. (**b**) Total bacterial 16 S rRNA gene copies and order analysis of fecal pellets from morphine-pelleted mice treated with vancomycin (VAN). The MP rRNA from Fig. 1 is presented here for comparison. Vancomycin treatment resulted in a significant reduction of Gram-positive (clostridiales and lactobacilliales), but not gram-negative (bacteroidales and enterobacteriales), bacteria. ns not significant, **p < 0.01, ****p < 0.0001 by unpaired Student’s t-test.

To determine which components of the antibiotic cocktail were the most influential in tolerance prevention, the time course of tolerance development was measured in mice treated with various combinations of antibiotics given by oral gavage. As shown in Fig. 8a, combinations that included vancomycin showed the most significant prevention of morphine tolerance. Vancomycin alone was sufficient to prevent tolerance, however this effect was less efficacious than the combination that included streptomycin, and ampicillin. The changes in bacterial 16 S rRNA from fecal samples of vancomycin-treated mice was compared to those of MP samples (Fig. 8b). While no significant changes in total bacterial 16 S rRNA was observed with vancomycin, there was a significant decrease in the percentage of Clostridiales and Lactobacilliales consistent with vancomycin’s effect on Gram-positive bacteria.

### Gut bacteria depletion does not affect morphine dependence

**Figure 9:**
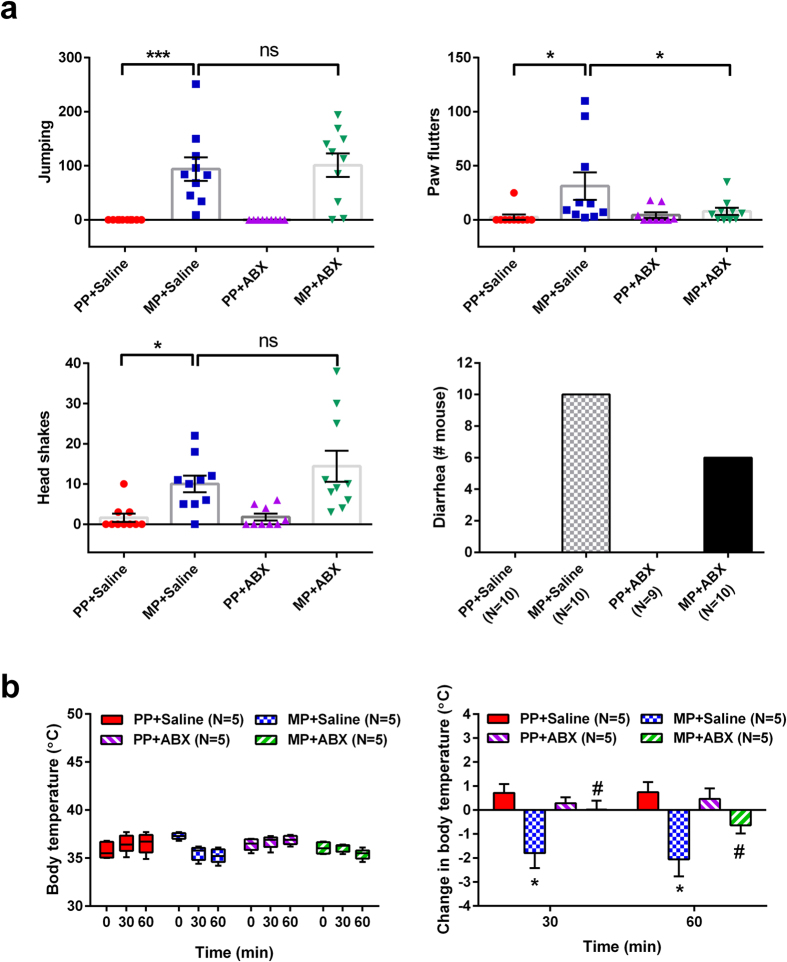
Antibiotic treatment does not alter chronic morphine-induced dependence. Withdrawal was precipitated by naloxone administration (1 mg/kg, s.c.) in all treatment groups. (**a**) Withdrawal behavior was scored by the presence of jumping behavior, paw flutters, head shakes, and diarrhea. ABX treatment did not significantly alter withdrawal precipitation following chronic morphine exposure. N = 10, ns not significant, *p < 0.05, **p < 0.01 by two-way ANOVA with Bonferroni post-hoc analysis. (**b**) Body temperature at 0, 30, and 60 min after naloxone administration (*left panel*). N = 5. Change in body temperature before and after naloxone administration (*right panel*). N = 5, *p < 0.05 (PP + SAL vs MP + SAL), ^#^p < 0.05 (MP + SAL vs MP + ABX) by two-way repeated-measures ANOVA with Bonferroni post-hoc analysis.

To address whether gut bacterial depletion alters morphine dependence, each treatment cohort was administered an acute naloxone injection (1 mg/kg, s.c.), and monitored for signs of precipitated withdrawal (jumping, paw flutters, head shakes, hypothermia, and diarrhea). Results are summarized in Fig. 9. Neither of the PP groups demonstrated significant modification of behavior with naloxone exposure. MP + SAL mice demonstrated significant increases in jumping (p < 0.001), paw flutters (p < 0.05) and head shakes (p < 0.05), reduced rectal temperature at 30 and 60 min (p < 0.05), and precipitated diarrhea in all mice. ABX treatment appeared to alter some aspects of naloxone withdrawal in morphine-pelleted mice, but not others. ABX did not affect the ability of naloxone to precipitate increases in jumping and head shakes, but reduced paw flutters. Precipitation of diarrhea was evident in 6 of 10 mice in the MP + ABX group. Rectal temperature was reduced at 60 min, but not 30 min. Collectively, these data suggest that depletion of gut bacteria does not affect morphine dependence.

### Opioid tolerance in sensory neurons from the dorsal root ganglia

**Figure 10:**
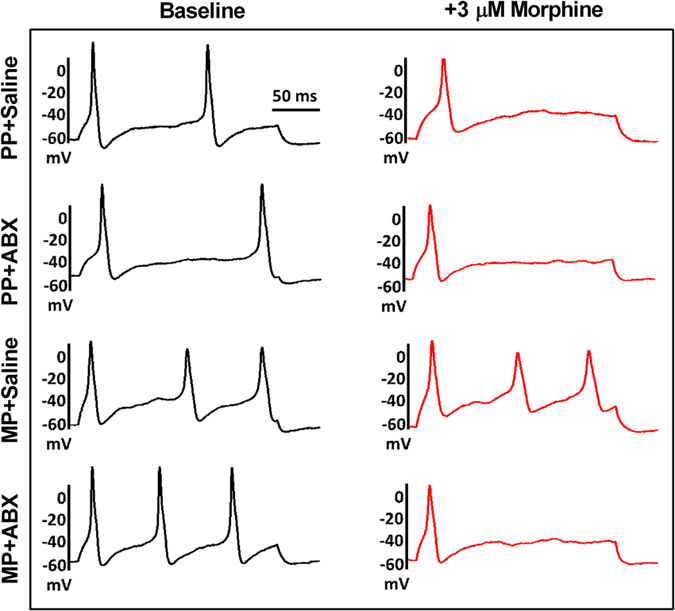
Morphine-induced reduction in electrical excitability of DRG neurons. Raw traces of action potentials elicited at double (2X) rheobase in DRG neurons from each treatment group. Application of morphine (3 uM) reduced the number of action potentials in all groups except MP + SAL, indicative of tolerance development.

**Figure 11:**
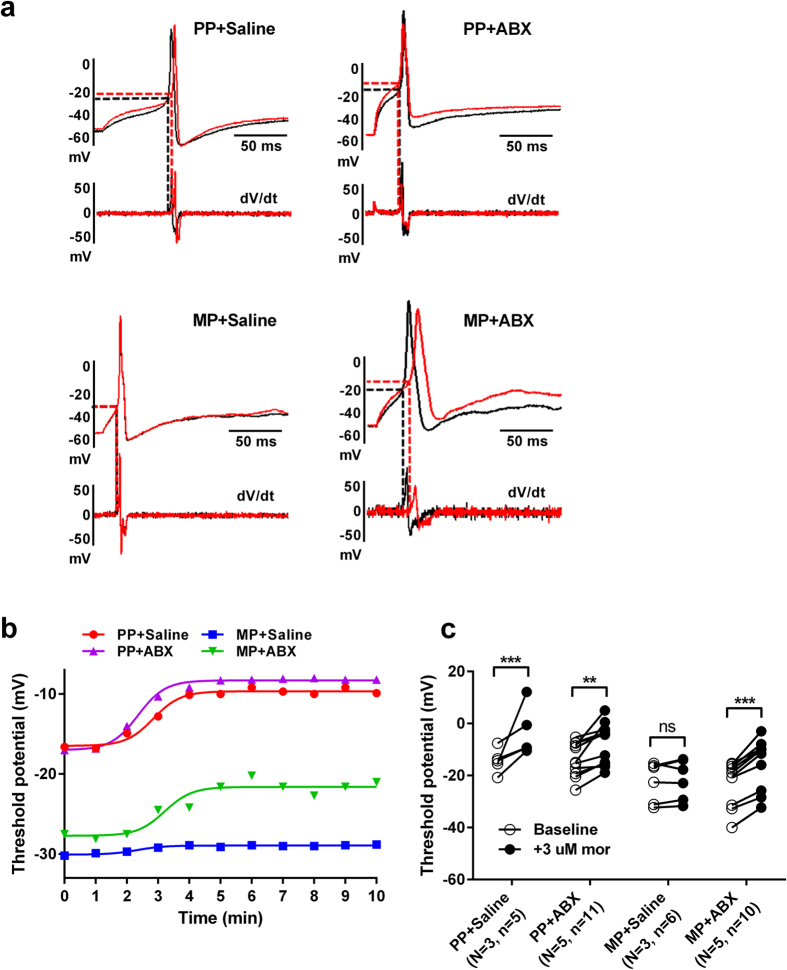
Antibiotic treatment prevents morphine tolerance in isolated neurons from dorsal root ganglia. (**a**) Raw traces of threshold potentials (V_t_) of DRG neurons from each treatment group. Increases in threshold potential (V_t_) induced by 3 μM morphine perfusion are prevented by chronic morphine administration, but preserved with ABX treatment. (**b**) Representative samples demonstrating the time-dependence of V_t_ responses to morphine perfusion for DRGs from each treatment group. (**c**) Collective data from V_t_ measurements in all treatment groups before (*open circle*) and after (*filled circle*) morphine perfusion. PP + SAL (N = 3, n = 5), PP + ABX (N = 5, n = 11), MP + SAL (N = 3, n = 6), MP + ABX (N = 5, n = 10), ns not significant, **p < 0.01, ***p < 0.001 by two-way repeated-measures ANOVA with Bonferroni post-hoc analysis.

To test whether the effects of bacterial depletion on morphine tolerance could be recapitulated in single cells, dorsal root ganglion (DRG) nociceptors were isolated from spinal levels supplying the lower alimentary canal (L5-S1). Active and passive properties of low capacitance (<30 pF) cells were measured from the DRG neurons of each group by whole-cell patch clamp techniques. A number of cell properties did not significantly vary between treatment groups, including membrane capacitance, series resistance, input resistance, and resting membrane potential (Table 1). However, cells from both MP + SAL and MP + ABX demonstrated increased excitability at baseline, manifested by reduced rheobases (current injection required to produce action potential) and threshold potentials (V_t_) (the potential at which the upstroke of the action potential occurs). Figure 10 shows raw traces of action potentials obtained at 2X rheobase from DRG neurons collected from each treatment group. Exposure to acute morphine resulted in reduced number of action potentials in neurons from PP + SAL, PP + ABX, and MP + ABX mice. However, acute morphine did not alter the excitability of neurons obtained from MP + SAL group, indicative of tolerance development. The reduced excitability is also seen as a shift in the threshold potential (Fig. 11). Neurons from PP + SAL, PP + ABX and MP + ABX responded to morphine challenge by a positive shift in V_t_ indicative of morphine-induced reduction in excitability. The threshold potential was determined from the derivative of the action potential (dV/dt) in the absence and presence of acute morphine challenge. Figure 11b shows the time course of the change in threshold potential with morphine exposure. Neurons from MP mice had lower thresholds irrespective of ABX treatment, however only the MP + SAL neurons did not respond to morphine challenge.

## Discussion

The mechanisms producing opioid tolerance have been studied for many decades given the clinical and social implications of physical dependence and addiction. Despite multiple studies at different levels in various tissues, no single regulatory mechanism can account for all variance noted in tolerance development. This reflects the fact that opioid tolerance is a complex phenomenon, involving modulation by multiple cellular pathways. Chronic morphine has recently been shown to alter the resident microbial composition of the gastrointestinal tract and induce bacterial translocation across the gut epithelial barrier through mechanisms involving toll-like receptor (TLR) 2 and 4^7^. In the present study, we found that bacterial depletion with broad-spectrum antibiotic treatment (ABX) prevented many of the pathologic changes occurring in the gut with chronic morphine treatment, including mucosal barrier permeability, destruction, and inflammation. Further, we demonstrate that significant reduction in the gut luminal bacteria with the use of broad-spectrum ABX prevents development of antinociceptive tolerance, one of the major limiting factors of clinical opioid use. Since oral vancomycin alone had substantial effect in the development of antinociceptive tolerance, is consistent with the thesis that dysbiosis of the gut microbiome, particularly vancomycin-sensitive Gram-positive bacteria, influence mechanisms associated with tolerance to chronic morphine. Our findings are also consistent with those of Meng *et al*.^7^ demonstrating a decrease in lactobacilliales and bacteroidales with an increase in enterobacteriales with chronic morphine. Others have also shown the expression of increased virulence of *Pseudomonas aeruginosa* in the gut in the presence of chronic morphine^12^.

Significant depletion of the gut bacteria population was achieved by oral gavage of a broad-spectrum antibiotic cocktail in addition to ampicillin supplementation of drinking water. Similar regimens have previously been demonstrated to replicate many of the features of axenic (germ-free) mice, including gross anatomic and immunologic phenotypes, such as cecal enlargement and splenic atrophy^11^,^13^,^14^. Chronic morphine also reduced spleen weight, possibly due to immune suppression. This may be further exaggerated in the absence of any bacterial translocation that may precipitate atrophy. While others have supplemented numerous antibiotics in drinking water, the resulting taste aversion is known to result in severe dehydration and weight loss. Oral gavage delivery provides a viable alternative to this strategy that circumvents dehydration and allows controlled dosing. A notable asset of drinking water supplementation, however, is continuous drug delivery. To maintain this advantage, we supplemented drinking water with ampicillin at a dose that did not produce significant weight loss or dehydration. This antibiotic treatment (ABX) began depleting gut bacterial load by day 4 of treatment, and resulted in almost 90% clearance in fecal pellets by day 8. Chronic morphine treatment resulted in enhanced gut permeability consistent with the disruption of the membrane localization of the tight junction protein, occludin, in the colon. This disorganization of occludin by chronic morphine at the tight junction was prevented with ABX treatment thus maintaining epithelial integrity as noted by the reduced FITC-dextran in serum. Interestingly, we also observed increased mRNA expression of occludin and ZO-1 in the chronic morphine treated mice with ABX (Supplemental Data). Various antibiotics have been shown to improve epithelial integrity in cystic fibrosis mice^15^, in stress-induced^16^ and an increase in the mRNA expression of tight junction proteins has been demonstrated with neomycin and bacitracin^17^. The specific mechanism by which mRNA increase occurs only after chronic morphine in the ABX treated group is unclear but may be related to the development of stress.

To test the effect of bacterial clearance on morphine tolerance, mice were implanted with morphine (MP) or placebo pellets (PP) on day 5 of ABX treatment. Results from behavioral tests of nociception (tail-immersion and acetic acid stretch assays) indicated that ABX prevented tolerance formation induced by chronic morphine treatment. These two behavioral paradigms were selected due to characteristic differences in the types of nociception they assess. The tail-flick response involves somatic afferents and a spinally-mediated reflex arc, while the acetic acid stretch assay recruits visceral afferents and supra-spinally-mediated neural pathways. Interestingly, there was a significant leftward shift in the response to morphine challenge following antibiotics. This is reflected in higher baseline latencies in the morphine-pelleted mice and the lack of tolerance development.

The dorsal root ganglia (DRG) contain neuronal soma for the afferents projecting from both somatic and visceral regions and serves as a “relay station” to the central nervous system. We^18^ and others^19^,^20^ have previously shown that nociceptive DRG neurons projecting from the colon are hyperexcitable following chronic morphine treatment, partly as a result of a shift in the activation of TTX-resistant sodium channels and enhanced TRPV1 expression. In the present study this is reflected in DRG neurons by the lower threshold potential in the morphine pelleted groups. However, while neurons from MP + SAL mice were tolerant to morphine-induced increases in threshold potential (V_t_), neurons from MP + ABX mice continued to respond to morphine, indicating a lack of tolerance. This finding concurs with the observations made in behavioral assays of nociception, and suggests a role of intestinal microbiota in altering the cellular mechanisms responsible for morphine tolerance in DRG neurons. Inflammation in the colon increases the excitability of sensory neurons projecting from the dorsal root ganglia^21^,^22^ due to an increase in TTX-resistant Na^+^ currents. It is therefore likely that chronic morphine-induced colonic inflammation secondarily also affects the excitability of DRG neurons and modulates the development of tolerance. The process of tolerance and desensitization occurs in a time-dependent fashion, whereby at short time scales receptor phosphorylation, β-arrestin2 recruitment, and receptor internalization may predominate. However, the mechanisms associated with long-term tolerance are not fully understood. Our findings that reducing gut bacteria affects tolerance suggest that bacterial translocation may be an important component in mediating long-term tolerance. Whether this is due to an acute inflammatory response or direct effects of bacteria remains to be determined. It is noteworthy, however, that certain bacteria including *Staphylococcus aureus* can directly activate sensory neurons via bacterial N-formylated peptides^6^, and that morphine significantly increases Staphylococcus in the gut lumen^23^.

An important question that arises from the use of antibiotics is whether the loss of tolerance is due to gut bacterial depletion/alteration or other off-target effects. The concentrations of morphine in the brain were similar in the absence and presence of ABX treatment, suggesting that ABX did not affect the distribution of morphine. Antibiotic pre-treatment prior to pellet implantation was necessary to prevent morphine tolerance. Removing this pre-treatment phase and implanting morphine pellets on the same day as initiation of ABX (i.e. at a time when bacterial load was unchanged), was insufficient to prevent tolerance development. It is also noteworthy that the concentration of antibiotic cocktail used in the present study was significantly lower than those used by Reikvam *et al*.^11^ and that the lack of tolerance to morphine persists after removal of the pellet *in vivo*, as well as in DRG neurons isolated and placed in culture. Moreover, of all combinations examined, the presence of vancomycin appeared to be necessary for the prevention of morphine antinociceptive tolerance. Vancomycin is not absorbed from the intestine and has 0% bioavailability when administered orally. The loss of antinociceptive tolerance to morphine with oral vancomycin alone suggests that the gut microbiome affects the development of tolerance. Our finding of reduced Lactobacilliales and Clostridales with vancomycin is consistent with the known effects of this antibiotic on Gram-positive bacteria. Although chronic morphine slightly reduced Gram-positive bacteria, tolerance may still be modulated by the Gram-positive dysbiosis occurring with morphine exposure (e.g. compositional shifts from authochtonous taxa, induction of virulence in various strains, or bacterial translocation). Further studies will be needed to define the specific changes in the bacterial population under chronic morphine conditions. Recent studies have shown significant dysbiosis in the fecal samples of cirrhotic patients taking opioids, with lower relative abundance of autochthonous taxa and Bacteroidaceae^24^. Thus, dysbiosis of the gut mcirobiome appears to be the primary mechanism for tolerance to the antinociceptive effects of chronic morphine. The specific mechanism by which the gut microbiome alters tolerance development remains to be determined, however, it is possible that breakdown of the epithelial barrier, microbial translocation and inflammation within the colonic wall may alter extrinsic sensory neurons thus leading to the development of tolerance.

The notion that bacterial products can affect opioid tolerance has been suggested from several previous studies. Initial work demonstrating that microglia and astrocyte activation occurs as a result of chronic morphine treatment has been followed by demonstration of opioid activation of the glial TLR4 receptor resulting in inflammatory responses and development of tolerance^25^,^26^,^27^. Furthermore, antagonists of TLR’s enhance the analgesic efficacy of opioids^27^. Collectively, these studies point toward an important role for the gut microbiota and bacterial derived products that activate TLRs in the development of opioid tolerance. Previous studies have shown that antibiotic treatment may also change the luminal and mucosal bacterial communities^11^,^28^. Whether specific changes in the composition of the bacteria regulates development of tolerance remains to be determined. The basis for the dysbiosis of gut microbiome by chronic morphine may be attributed to the slowing of colonic transit as a result of direct effects of morphine on enteric neurons^29^. Indeed, the gut mucosal microbiome is altered in patients with constipation^30^. In addition, morphine induces spasms of the Sphincter of Oddi thus affecting bile acid secretion into the duodenum. It is unclear if this leads to changes in the gut microbiome. However, patients with liver cirrhosis demonstrate significant changes in the gut bacterial population with an increase in fecal secondary bile acids and inflammation in colonic tissues^31^. Bile acids can directly affect colonic motility and therefore may lead to dysbiosis. Further work is needed to define the basis for the dysbiosis, nevertheless the findings of this study implies a significant role for the gut-brain axis through modulation of gastrointestinal bacteria on opioid tolerance.

## Methods

### Animals

Male Swiss Webster mice (Harlan Sprague Dawley, Inc. Fredrick, MD, USA) weighing 25–30 g were housed five to a cage in animal care quarters under a 12-hour light/dark cycle with food and water available *ad libitum*. All animal procedures were conducted in accordance with the procedures reviewed and approved by the Institutional Animal Care and Use committee at Virginia Commonwealth University (VCU IACUC).

### Group sizes

The sample size “N” for each experimental condition is provided in the results and figure legends. These values represent independent observations, not replicates. For electrophysiology experiments, “N” represents the total number of animals, and “n” the total number of cells. Data are represented in a scatter plot for individual animals defining the distribution of the responses. For time course studies and dose-response relationships, the data are presented as line graphs. Based on previous assessments of the reproducibility of morphine pellet-induced opioid tolerance^18^,^29^,^32^,^33^,^34^, a sample size of 5 was used to generate the dose-response relationship. For time-course studies assessing the effect of antibiotics on opioid tolerance, the sample size was 10.

### Randomization

All animals were randomly divided into control and treatment groups.

### Blinding

No blinding was performed in these experiments. The effect of the antibiotics on tail-flick latency was, however, performed by two separate investigators to obtain reliability of the effect.

### Replication

The qPCR data for IL-1β are presented as means of biological replicates (N = 6), and not technical replicates (n = 2), for each sample point.

### Antibiotic treatment

Broad-spectrum antibiotic treatment involved oral gavage of streptomycin (10 mg/kg), neomycin (10 mg/kg), vancomycin (5 mg/kg), and metronidazol (10 mg/kg) every 12 hours (modified from^11^,^35^,^36^, Sigma-Aldrich, St. Louis, MO). Drinking water was additionally supplemented with ampicillin (1 g/L, Sigma-Aldrich, St. Louis, MO) and available *ad libitum*. Control mice received saline gavage treatments every 12 hours and normal drinking water (Fig. 2). In separate experiments, different combinations of antibiotics were provided by oral gavage (Fig. 8).

### Chronic morphine treatment

For chronic morphine administration, a 75 mg morphine or placebo pellet was implanted subcutaneously on the dorsum. Mice were anesthetized with 2.5% isoflurane before shaving the hair from the base of the neck. The skin was cleansed with 10% povidone iodine (General Medical Corp., Walnut, CA) and rinsed with alcohol before making a 1 cm horizontal incision at the base of the neck. The subcutaneous space was opened by insertion of a sterile glass rod toward the dorsal flanks. The pellet was inserted in the space before closing the site with Clay Adams Brand, MikRon AutoClip 9-mm Wound Clips (Becton Dickinson, Franklin Lakes, NJ) and cleansing the surgical site again with 10% povidone iodine. Maintenance of a stringent aseptic surgical field minimized any potential contamination of the pellet, incision, and subcutaneous space. The animals were allowed to recover in their home cages where they remained throughout the experiment. Mice were sacrificed at the end of the 10 day ABX treatment.

### FITC-dextran assay

For *in vivo* intestinal permeability studies, FITC-conjugated dextran (Sigma-Aldrich, St. Louis, MO) or saline was administered by oral gavage (44 mg/100 g body weight of FITC-labeled dextran) 4 hours before whole blood collection by cardiac puncture. Plasma was isolated from blood samples by centrifugation for 15 min at 3000 rpm and 4 °C. FITC concentration was fluorometrically quantified by emission spectrometry (Promega, Madison, WI) at 528 nm, using an excitation wavelength of 485 nm. All concentrations were measured against a standard curve of serially diluted FITC-dextran.

### Assessment of bacterial translocation

To assess bacterial translocation following chronic morphine exposure, colon, mesenteric lymph node (MLN), liver, and spleen samples were collected from mice receiving morphine pellets. Tissues were homogenized 1:10 in phosphate-buffered saline (PBS) containing 0.1% Tween 20 per gram of tissue, then diluted again 1:10 in PBS. Diluted homogenates were plated on blood agar plates and incubated at 37 °C for 1–2 days. Colony forming units (CFU) were counted, and densities calculated as follows: CFU/mL = (# of colonies × sample dilution factor × serial dilution factor)/volume of culture plate (mL).

### Quantification of bacterial 16S rRNA gene

Fecal samples were collected from mice, immediately snap frozen in liquid nitrogen, and stored at −80 °C. Microbial genomic DNA was extracted with QIAamp DNA Stool Kit (QIAGEN, Germantown, MD) according to the manufacturer’s protocol, and used for the amplification of the 16 S rRNA gene using group-specific primers as shown in Table 2. The reaction mixture (20 uL) contained 200 nM forward primer, 200 nM reverse primer, 10 μL of 2X SuperMix buffer, 1 μL of BSA (10 μg/μL), and 20 ng total DNA. With the use of the iTaq Universal SYBR Green SuperMix (Bio-Rad, Hercules, CA) kit, PCR tubes were incubated with an enzyme activation step for 3 min at 95 °C. The PCR protocol consisted of 40 cycles of denaturation (15 s at 95 °C), annealing and extension (15 sec at 60 °C). 16 S rRNA gene abundance in fecal samples was determined by comparing the cycle threshold (Ct) values to those generated by a standard curve. The standard curves were developed by applying the group-specific primers to DNA purified from cultured American Type Culture Collection (ATCC) (Manassa, VA) strains of bacteria. Each bacterial gene copy number was then calculated from total DNA concentration for each PCR product (www.scienceprimer.com) and expressed as 16 S rRNA gene copies per gram of wet weight.

### Hematoxylin and eosin (H&E) staining

Colons samples were isolated from all treatment groups, and immediately placed in carbogen (95% O_2_/5% CO_2_)-bubbled ice-cold Krebs solution containing (in mM): 118 NaCl, 4.6 KCl, 1.3 NaH_2_PO_4_, 1.2 MgSO_4_, 25 NaHCO_3_, 11 glucose, and 2.5 CaCl_2_. Fecal material was gently flushed with Krebs solution. The tissue was embedded in Optimal Cutting Temperature (OCT) Compound (Sakura Finetek, Torrance, CA) and cyrosectioned at 20-μm intervals. The cross-sections were fixed in 4% paraformaldehyde in PBS and stained by standard hematoxylin and eosin (H&E, Sigma-Aldrich, St. Louis, MO) protocol.

### Immunohistochemical staining

Colon samples were isolated from all treatment groups. For immunostaining, 20 μm frozen sections were fixed with 4% paraformaldehyde in PBS for 20 min at room temperature. After washing in PBS and permeabilization with Triton X-100 (0.1%) and blocking nonspecific binding sites with 5% goat serum, tissues were incubated with mouse anti-occludin at 2 ug/mL (Invitrogen, Carlsbad, CA) in PBS with 5% goat serum overnight at 4 °C. After washing in PBS, sections were incubated with goat anti-mouse IgG (H + L), Alexa Fluor 488 conjugated secondary antibody (Life Technologies, Carlsbad, CA) at 2 ug/mL for 2 h at room temperature. Sections were then washed and mounted under coverslips using ProLong Gold antifade reagent with DAPI (Life Technologies, Carlsbad, CA). Sections were imaged using a confocal microscope (Nikon).

### Nociceptive response tests

To test nociceptive response to morphine, the tail-immersion and acetic acid stretch assays were used in this study. In the tail-immersion assay, mice were gently detained in a cloth, and the distal 1/3 of the tail immersed in a water bath at 52 °C. The latency to tail-flick was recorded, with a maximum latency of 10 sec (to prevent tissue damage). In the acetic acid stretch assay, mice were injected with 0.6% acetic acid (10 μL/g body weight, i.p.) and transferred to a testing chamber. Following a 3 min interval for onset of action, the number of stretches were recorded for 15 min. In both assessments acute morphine was administered (10 mg/kg, s.c.) 25 min prior to testing. Where indicated, morphine antinociception in the tail-immersion assay was quantified as the percentage of maximum possible effect (%MPE), such that: %MPE = [(challenge time − baseline time)/(10 − baseline time)] × 100.

### RNA isolation and real-time PCR

Quantitative real-time polymerase chain reaction (qRT-PCR) was performed on a Mini-Opticon real-time PCR system (Bio-Rad, Hercules, CA). Ribosomal 18 S RNA was used as an internal control. Sample tissues were isolated, and total RNA was extracted using TRIzol reagent (ThermoFisher Scientific, Waltham, MA). The first-strand cDNA synthesis was amplified at 42 °C for 30 min and subsequent polymerization was performed in a single step using the SensiMix One-Step kit (Bio-Rad, Hercules, CA). The reaction mixture (20 uL) contained 200 nM forward primer, 200 nM reverse primer, 1 uL of 2X SensiMix One-Step buffer, 10 units RNase inhibitor, and 200 ng total RNA. With the use of the one-step SensiMix kit, reverse transcription was performed for 30 min at 42 °C with an enzyme activation step for 10 min at 95 °C. The PCR protocol consisted of 40 cycles of denaturation (15 sec at 95 °C), annealing (30 s at 58 °C), and extension (30 sec at 72 °C). Relative expression of the respective genes to 18 S expression was calculated using the ΔΔCt method and values were expressed as fold change from placebo animal. Primers were in this study used shown in Table 2. The Il-1β mRNA was measured from the colons obtained at day 1, 3, 5, 7 after morphine or placebo pellet implantation.

### Quantification of brain morphine

The primary reference materials of morphine-d3 (the internal standard) and morphine were purchased from Cerilliant Corporation (Round Rock, Texas) as metabolic solutions. The chloroform, deionized (DI) water, hydroxamine hydrochloride (HCL), 2-propanol, sodium bicarbonate and sodium carbonate were purchased form Fisher Scientific (Hanover Park, Illinois). BSTFA (N, O-bis(trimethylsilyl)-trifluoroacetamide) +10% TMCS (Trimethylchlorosilane) was purchased from Regis Technologies (Morton Grove, Illinois). Quantitative analysis of morphine was based upon a previously described method (1, 2). In brief, whole brain tissues specimens were diluted as 1:3 in DI water and homogenized. A five-point calibration curve of 20–1000 ng/g morphine in 1.0 g of drug-free mouse tissue homagate, a blank control and a double blank control were prepared. 50 μL of ISTD consisting of 10 μg/mL (500 ng total) of morphine-d3 was added to 1.0 g aliquots of calibrators, quality control (QC) specimens or sample homogenates except the double blank control. 0.2 mL of 10% hydroxamine HCL was added to each sample. They were then mixed and heated at 30 °C for 30 min. Samples were then cooled and 1 mL of saturated carbonate/bicarbonate buffer (1:1, N:N, pH 9.5) and 3 mL of chloroform:2-propanol (9:1) were added. Samples were mixed for 5 min and centrifuged at 2500 rpm for 5 min. The top aqueous layer was aspirated and the organic layer transfered to a clean test tube and evaporated to dryness at 40 °C under a constant stream of nitrogen. 50 μL of BSTFA + 10% TMCS was added and the samples were heated for 30 min at 70 °C. The samples were then placed in auto-sampler vials for gas chromatography mass spectrometry (GC/MS) analysis. The GC/MS analysis was performed with a Hewlett-Packard 6890 with a split/splitless injection port attached to Hewlett-Packard model 5793 A mass selective detector (MSD) with a 7683 autosampler. The chromatographic separation was preformed on an Agilent (Santa Clara, CA) HP-1 12 mm × 0.2 mm × 0.33 μM analytical column with the injection temperature set to 170 °C and run in pulsed splitless mode. The initial oven temperature was 170 °C and was held for 1 min then heated at 10 °C/min to 280 °C. The total run time was 12 min. The quantification and qualifying ions monitored for morphine were 236, 414 and 429 m/z and for morphine-d3 they were 239 and 432 m/z. A linear regression of the ratio of the peak area counts of quantification of morphine and morphine-d3 versus concentration was used to construct the calibration curves.

### Naloxone-precipitated withdrawal

Withdrawal susceptibility was assessed by administration of the selective μ-opioid receptor inverse agonist naloxone (1 mg/kg, s.c.) Following injection, mice were monitored for 20 min in acrylic chambers for signs of withdrawal precipitation (jumps, paw flutters, head shakes, hypothermia, and diarrhea). Rectal temperature was assessed at baseline, 30 min, and 60 min after naloxone administration using a digital thermometer. Animals were handled during the period of acclimation before initiating experiments to minimize the effect of stress on body temperature measurements.

### DRG isolation

Dorsal root ganglia were harvested from spinal levels supplying the distal alimentary canal (L5-S1; modified from Malin *et al*., 2007) and immediately placed in cold (4 °C) Hanks’ Balanced Salt Solution (HBSS, ThermoFisher Scientific, Waltham, MA). Ganglia were incubated (37 °C) for 18 min in HBSS with 15 U/mL papain, washed, and incubated for 1 hr in HBSS with 1.5 mg/mL *Clostridium histolyticum* collagenase. Tissues were gently triturated and centrifuged for 5 min at 1,000 rpm. The supernatant was decanted, and cells resuspended in neurobasal A medium containing 1% fetal bovine serum, 1× B-27 supplement, 10 ng/mL glial cell line-derived neurotropic factor (GDNF, Neuromics, Edina, MN), 2 mM L-glutamine (ThermoFisher Scientific, Waltham, MA), and penicillin/streptomycin/amphotericin B. The suspension was plated on poly-D-lysine- and laminin-coated coverslips (ThermoFisher Scientific, Waltham, MA) and incubated for 24 h.

### Electrophysiology

Coverslips were transported to a microscope stage plate and continuously superfused with external physiologic saline solution (PSS) containing (in mM) 135 NaCl, 5.4 KCl, 0.33 NaH_2_PO_4_, 5 HEPES, 1 MgCl_2_, 2 CaCl_2_, and 5 glucose. A GΩ seal was achieved via pulled (Model P-97 Flaming/Brown Micropipette Puller, Sutter Instruments, CA) and fire-polished (2–4 MΩ) borosilicate glass capillaries (World Precision Instruments, Sarasota, FL) filled with internal PSS containing (in mM) 100 L-aspartic acid (K salt), 30 KCl, 4.5 Na_2_ATP, 1 MgCl_2_, 10 HEPES, and 0.1 EGTA. DRG nociceptors being of the small, C and Aδ fiber types (Jin *et al*., 2013), only low capacitance ( < 30 pF) neurons were selected for analysis. Standard patch clamp techniques were performed using an Axopatch 200B amplifier (Molecular Devices, Sunnyvale, CA) and associated Clampex and Clampfit 10.2 software. Current clamp step protocols consisting of a 0 nA resting current and 0.01 nA steps from −0.03 nA were employed to assess passive and active cell properties. Taking the derivative of the voltage with respect to time (dV/dt), threshold potentials were defined as the voltage at which dV/dt significantly deviated from zero in the course of an action potential uprise. For morphine tolerance studies, external PSS was supplement with 3 μM morphine and threshold potentials recorded at 1 min intervals for 10 min. The maximal effect of morphine was recorded for each cell.

### Statistical analysis

Statistical differences were calculated by Student’s two-tailed t-test using GraphPad Prism 6.0 (GraphPad Software, Inc., La Jolla, CA). For group comparison, results were calculated by two-way ANOVA with Bonferroni post-hoc analyses. Differences were considered significant if p < 0.05. The results are expressed as mean value ± SEM.

## Additional Information

**How to cite this article**: Kang, M. *et al*. The effect of gut microbiome on tolerance to morphine mediated antinociception in mice. *Sci. Rep.* **7**, 42658; doi: 10.1038/srep42658 (2017).

**Publisher's note:** Springer Nature remains neutral with regard to jurisdictional claims in published maps and institutional affiliations.

## Supplementary Material

Supplementary Figure 1

## Figures and Tables

**Table 1:** Electrophysiological properties of DRG neurons.

	PP + Saline	+ 3 uM Morphine	PP + ABX	+ 3 uM Morphine	MP + Saline	+ 3 uM Morphine	MP + ABX	+ 3 uM Morphine
CMem (pF)	18.5 ± 1.6		16.1 ± 1.0		15.8 ± 1.9		17.4 ± 1.9	
Rseries (MΩ)	8.6 ± 0.8		9.5 ± 1.0		9.1 ± 0.8		8.8 ± 0.5	
VRest (mV)	−58.2 ± 1.6	−57.0 ± 2.1	−55.5 ± 1.1	−56.0 ± 1.0	−58.8 ± 2.8	−59.8 ± 2.8	−57.0 ± 2.4	−54.5 ± 2.3
AP VThresh (mV)	−13.2 ± 2.1	−3.4 ± 4.3***	−12.5 ± 1.8	−7.8 ± 2.4**	−20.7 ± 2.3	−21.6 ± 3.2	−23.1 ± 2.0	−15.5 ± 3.1**
AP VPeak (mV)	51.6 ± 6.0	53.4 ± 5.6	56.2 ± 5.5	61.1 ± 5.9	43.3 ± 6.4	58.0 ± 3.4	46.6 ± 4.1	49.4 ± 2.5
AP Amp (mV)	64.8 ± 5.0	56.8 ± 4.0	71.5 ± 4.9	72.6 ± 5.3	60.0 ± 9.5	82.1 ± 9.3	70.1 ± 4.6	64.3 ± 4.0
Rheobase (nA)	0.15 ± 0.06	0.21 ± 0.07	0.17 ± 0.04	0.22 ± 0.05	0.05 ± 0.02	0.03 ± 0.01	0.05 ± 0.01	0.07 ± 0.01
RInput (MΩ)	479.4 ± 158.3	257.5 ± 62.8	741.3 ± 222.9	662.5 ± 199.5	422.8 ± 132.2	671.7 ± 41.67	286.7 ± 66.7	468.3 ± 168.9

**Table 2:** List of primers used for quantification of bacterial specific 16 S rRNA and IL-1β.

Target	Sequence	Amplicon (bp)
IL-1β	Forward: 5′-CCTGAACTCAACTGTGAAATGC-3′	143
	Reverse: 5′-CGAGATTTGAAGCTGGATGC-3′	
18 S	Forward: 5′-TCAAGAACGAAAGTCGGAGG-3′	488
	Reverse: 5′-GGACATCTAAGGGCATCAC-3′	
Bacteroidales^1)^	Forward: 5′-GGTGTCGGCTTAAGTGCCAT-3′	151
	Reverse: 5′-CGGAYGTAAGGGCCGTGC-3′	
Clostridiales^2)^	Forward: 5′-CGGTACCTGACTAAGAAGC-3′	429
	Reverse: 5′-AGTTTYATTCTTGCGAACG-3′	
Lactobacilliales^3)^	Forward: 5′-GGAAACRRWGCTAATACCG-3′	189
	Reverse: 5′-GAAGATTCCCTACTGCT-3′	
Enterobacteriales^4)^	Forward: 5′-ATGGCTGTCGTCAGCTCGT-3′	177
	Reverse: 5′-CCTACTTCTTTTGCAACCCACTC-3′	
Universal total bacterial 16 S rRNA^5)^	Forward: 5′-ACTCCTACGGGAGGCAGCAGT-3′	180
	Reverse: 5′-ATTACCGCGGCTGCTGGC-3′	
Occludin	Forward: 5′-TCTATAAGTCAACACCTCTGGTGCC-3′	304
	Reverse: 5′-TGCAGACCTGCATCAAAATTTCTC-3′	
Zonula occludens-1a (ZO-1)	Forward: 5′-AAGAAGGCTTAGAGGAAGGTGATCA-3′	278
	Reverse: 5′-GTATAAAGTATCCACGACCCGGAA-3′	

The following type strains were used as positive controls: 1) B. Fragilis (ATCC 25285D-5), 2) B. Producta (ATCC 27340D-5), 3) L. Delbrueckii (ATCC 11842D-5), 4) and 5) E. coli (ATCC 700926D-5)
