# Supplementary material for: The effect of gut microbiome on tolerance to morphine mediated antinociception in mice
Source: Sci Rep. 2017 Feb 17;7:42658. doi: 10.1038/srep42658 (PMC5314392; doi:10.1038/srep42658)
Supplement: Supplementary Figure 1 [file srep42658-s1.pdf]

The effect of gut microbiome on tolerance to morphine mediated antinociception in mice

Minho Kang, Ryan A. Mischel, Sukhada Bhave, Essie Komla, Alvin Cho, Charity Huang, William L. Dewey, Hamid I. Akbarali

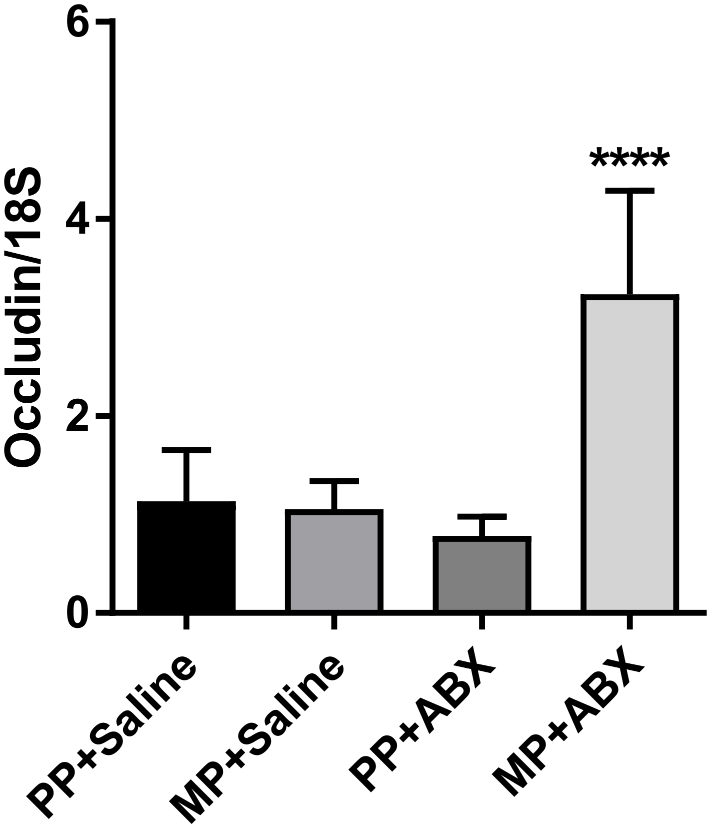

Occludin/18S
6
4
2
0
****
PP+Saline
MP+Saline
PP+ABX
MP+ABX

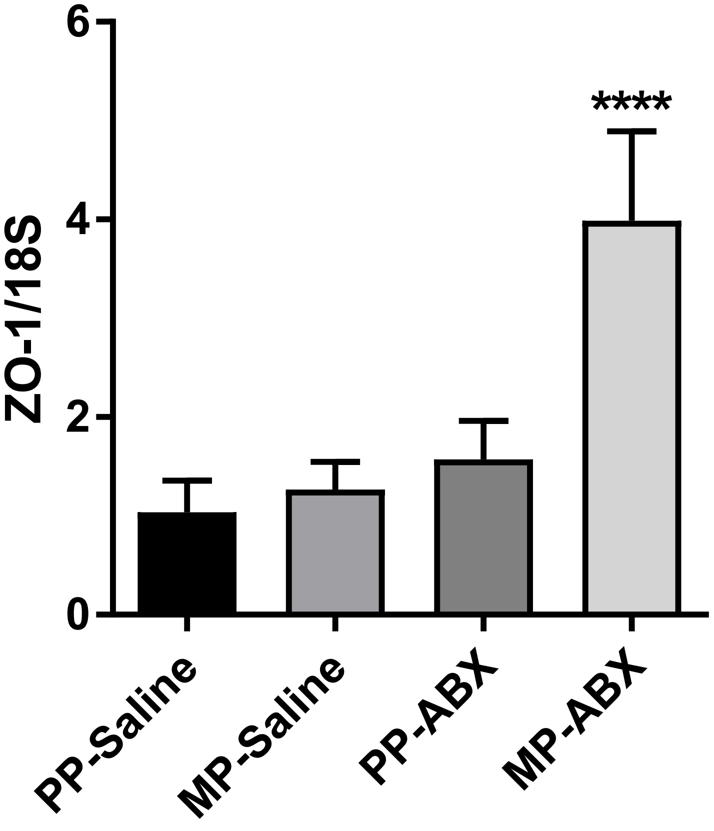

ZO-1/18S
6
4
2
0
****
PP-Saline
MP-Saline
PP-ABX
MP-ABX

Supplemental Figure 1

mRNA expression of occludin (top) and ZO-1 (bottom) in placebo and morphine pelleted mice colon treated with saline or antibiotics (ABX). The changes in expression were normalized to 18S RNA. N=5 ***$p<0.0001$ by two-way ANOVA with Bonferroni post-hoc analysis.
